# Which chronic obstructive pulmonary disease care recommendations have low implementation and why? A pilot study

**DOI:** 10.1186/1756-0500-5-652

**Published:** 2012-11-23

**Authors:** Kylie Johnston, Karen Grimmer-Somers, Mary Young, Ral Antic, Peter Frith

**Affiliations:** 1 International Centre for Allied Health Evidence, Division of Health Sciences, University of South Australia, Adelaide, Australia; 2 Transitional and Community Service, Royal Adelaide Hospital, Adelaide, Australia; 3 Department of Thoracic Medicine, Royal Adelaide Hospital, Adelaide, Australia; 4 Respiratory, Allergy and Sleep Services, Repatriation General Hospital and Flinders University, Adelaide, Australia

**Keywords:** Implementation, Guidelines, Chronic obstructive pulmonary disease, Pulmonary rehabilitation

## Abstract

**Background:**

Clinical care components for people with COPD are recommended in guidelines if high-level evidence exists. However, there are gaps in their implementation, and factors which act as barriers or facilitators to their uptake are not well described. The aim of this pilot study was to explore implementation of key high-evidence COPD guideline recommendations in patients admitted to hospital with a disease exacerbation, to inform the development of a larger observational study.

**Methods:**

This study recruited consecutive COPD patients admitted to a tertiary hospital. Patient demographic, disease and admission characteristics were recorded. Information about implementation of target guideline recommendations (smoking cessation, pulmonary rehabilitation referral, influenza vaccination, medication use and long-term oxygen use if hypoxaemic) was gained from medical records and patient interviews. Interviews with hospital-based doctors examined their perspectives on recommendation implementation.

**Results:**

Fifteen patients (aged 76(9) years, FEV_1_%pred 58(15), mean(SD)) and nine doctors participated. Referral to pulmonary rehabilitation (5/15 patients) was underutilised by comparison with other high-evidence recommendations. Low awareness of pulmonary rehabilitation was a key barrier for patients and doctors. Other barriers for patients were access difficulties, low perceived health benefits, and co-morbidities. Doctors reported they tended to refer patients with severe disease and frequent hospital attendance, a finding supported by the quantitative data.

**Conclusions:**

This study provides justification for a larger observational study to test the hypothesis that pulmonary rehabilitation referral is low in suitable COPD patients, and closer investigation of the reasons for this evidence-practice gap.

## Background

Clinical guidelines provide recommendations for chronic obstructive pulmonary disease (COPD) diagnosis and management based on published evidence. There is a high degree of agreement between international [1] and national COPD guidelines [2-6] supporting the efficacy of smoking cessation, pulmonary rehabilitation, influenza vaccinations, use of medications and long-term oxygen in hypoxaemic patients. However, there is a growing body of information to suggest that evidence-practice gaps regularly occur in COPD management [7]. Recent studies examining the extent and nature of such gaps have focused on the use of spirometry in COPD diagnosis, and guideline-based prescribing practices, using surveys [8] or medical record review [9].

However, few studies have examined the implementation of pulmonary rehabilitation in patients with COPD. Pulmonary rehabilitation includes exercise training, education and psychosocial support [10]. Pulmonary rehabilitation improves exercise capacity and health-related quality of life in patients with moderate-severe COPD, and reduces dyspnoea, anxiety, depression and health care utilisation [10,11]. A systematic review examining implementation of pulmonary rehabilitation in COPD patients found referral by primary care physicians to programs was low (3-16% of suitable COPD patients [12]). Few studies have combined prospective quantitative data with qualitative reporting to explore reasons for low implementation. Characteristics of COPD patients who do, and do not, receive guideline based care are largely unknown. The range and complexity of issues which constrain implementation of COPD guidelines require further investigation. Clarification of these issues will build a research platform upon which effective strategies for better guideline implementation can be developed.

The aims of this pilot study were, firstly, to evaluate expected versus actual clinical practice regarding implementation of key evidence-based recommendations for COPD management, in a consecutive sample of patients admitted to hospital with an acute exacerbation. Our intention was to explore which recommendations had low implementation, to inform the development of a subsequent larger observational study [13]. Secondly, where expected and actual practices differed, we aimed to conduct a preliminary exploration of barriers and facilitators to implementation for patients and doctors. This would inform our choice of interview questions for a substantive qualitative study.

Five guideline recommendations (supported by systematic reviews of high-quality randomised controlled trials [1]) were examined for evidence of their implementation. These comprised smoking cessation, referral to pulmonary rehabilitation, influenza vaccination, long term oxygen use if hypoxaemic, and guideline based medication use.

## Methods

Quantitative and qualitative methods and analysis were used to address the research questions. Ethical approval to conduct this study was obtained from the University of South Australia Human Research and Ethics Committee and the Royal Adelaide Hospital Human Ethics Committee prior to commencement.

### Patient data

Patients admitted to a metropolitan tertiary hospital with a documented primary condition of exacerbation of COPD, were invited to participate. Patients were identified from daily hospital admission lists. Patients were excluded if they were non-English speaking, unable to provide written informed consent, or physically not well enough to participate. Informed consent was obtained from all patients prior to participation in the study. In this pilot study patient admissions over a two month period were examined, rather than a pre-determined sample size. It was anticipated this would be sufficient to provide useful information about recruitment rate, and highlight which guideline recommendations were poorly implemented (difference of at least 50% between highest and lowest implementation).

Data collection included:

1. Medical record review, which extracted data on patient demographics, disease severity, co-morbidities, smoking status, current and previous admissions, and documented evidence of target COPD care recommendations being implemented.

2. Semi-structured face-to-face interview toward the end of hospital admission. Information about patients’ experience of, and perceived barriers and facilitators to, the target COPD care recommendations was sought.

### Medical practitioner data

For all recruited patients, contact was made with the hospital-based medical practitioner providing their inpatient care (i.e. general medical registrar or intern) at the end of the patient’s hospital admission. Informed consent to participate in the study was obtained from these health care providers. Semi-structured interviews were conducted with medical practitioners regarding their perspectives on implementation of target COPD recommendations, and barriers and facilitators to this process.

### Data analysis

#### Quantitative analyses

Frequencies and percentages of compliance with COPD recommendations were reported.

#### Qualitative analysis

Interviews with participants were audiotaped, transcribed verbatim, and the transcripts were content analysed to identify and classify themes. Thematic analysis of interviews focused on experience of, and barriers and facilitators to, implementation of target COPD recommendations. This process involved:

1. Identifying transcript excerpts which related to the research question for each COPD care guideline.

2. Inductive open coding to organise these groups of excerpts into themes.

3. Comparing themes and excerpts with existing analyses of barriers and facilitators to evidence-based health care implementation. To avoid being restricted to any one behaviour change theory, a consensus based model of theoretical domains for investigating evidence-based practice was used [14].

4. Ongoing adjustment of excerpts and themes; re-reading transcripts for further relevant data.

5. Semi-quantitative analysis to determine frequency of themes for each COPD care guideline.

Study rigour was enhanced by adherence to standardised data collection protocol including the semi-structured interview guide, transcription by an independent typist, and subsequent validation by the interviewer. An independent analyst experienced in qualitative research reviewed 20% of the transcript pages (from three interviews which were randomly selected) to identify themes from the data. Differences were contested through discussion and consensus reached. Our aim in this pilot study was not to gain data saturation but to highlight predominant themes in relation to implementation of this broad range of guideline recommendations, and to guide interview question development.

## Results

**Figure 1:**
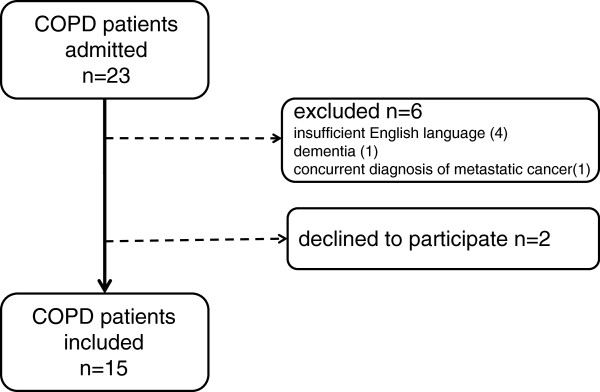
Recruitment flow chart.

Fifteen patients with COPD joined the study (from 23 consecutive admissions during recruitment period, Figure 1), a rate of 1.7 patients per week. Nine hospital doctors (5 registrars, 4 interns) were invited to participate in this study and all agreed. Characteristics of the COPD patient sample are shown in Table 1. Implementation of COPD guidelines at time of hospital admission is summarised in Table 2. Compared to recommedations with high implementation (e.g. influenza vaccination in 14/15 patients and guideline based medication use in 15/15 patients), referral to pulmonary rehabilitation was implemented in only 5 of 15 patients. Therefore, only associations with this recommendation are further described.

**Table 1:** Characteristics of the COPD patient sample

**Sample characteristics**	**Value**
Females, (%)	5 (33%)
Age, mean(SD)	75.8 (9.0) years
FEV_1_%	58.2 (15.2)%
COPD severity (GOLD criteria^1^)	
Moderate	7 (47%)
Severe	8 (53%)
Living arrangements	
Lives alone	7
Lives with partner	6
Lives with dependents (sole carer)	1
Lives in aged care facility	1
Current smokers	3
Home oxygen in use	2
Previously seen by a respiratory physician	6
Use of home support services	
No assistance other than spouse/relatives	9
Domestic assistance	4
Self-care assistance	2
Number of admissions for COPD last year, mean(SD)	2.5 (1.5)
Length of stay this admission, mean(SD)	6.7 (5.7) days
Acute hospital bed days in the last 3 years, median (25^th^ -75^th^ centile)	15 (7–27.5) days
Interventions during current admission	
COPD coordinator^*^	9/15 patients
Physiotherapist	12/15
Occupational therapist	2/15
Admission to intensive care unit^†^	1/15
New referral to respiratory physician	2/15

SD, standard deviation.

^*^ In this facility the COPD co-ordinator was a senior respiratory nurse, whose responsibility was to assist the medical teams to provide co-ordinated care for COPD patients.

^†^For bi-level positive pressure ventilation and cardioversion.

**Table 2:** Implementation of COPD guidelines at the time of hospital admission

	**Was recommendation met?**	**Compliance with recommendation (%)**	**Strategies used (frequency)**	**Involved staff members (frequency)**
**Smoking cessation**	Yes in 2 of 3 current smokers	67%	Encouraged use of quit line (2)	COPD-c (2)
			Prescribed transdermal nicotine patches (1)	medical (1)
**Pulmonary rehab referral**	Yes in 5 of 15	33%	Discussed with patient in 9/15 cases (60%)	COPD-c (8)
				medical (1)
**Flu vaccination**	Yes in 14 of 15	93%	Confirmed already completed by GP in 12/13 cases.	medical and COPD-c (14)
			Discussed with 1 patient who had not previously received it. Not discussed in one patient	
**Medication**	Yes in 15 of 15	100%	Prescribed medications checked by pharmacist (15)	pharmacy medical and COPD-c (13)
			Inhaled tiotropium added if not previously prescribed with (2 of 2 cases).	
			Reported non-use of spacer device with MDI in 6 cases (reported use in 5 cases, not required (2), unknown (2)	
**Long term home oxygen if hypoxaemic**	Yes in 5 of 5 cases	100%	Currently in place (2)	medical (5)
			Investigated by medical staff on wards this admission, for follow-up when patient stable (2)	
			Investigation planned to follow in outpatients (1)	

COPD-c = COPD co-ordinator; MDI = metered dose inhaler.

Patients who were referred to pulmonary rehabilitation at the time of the current admission had more severe lung disease (FEV_1%pred_ = 41.2[9.7], mean[SD], compared with FEV_1%pred_ = 60[13.7] in not referred group, p = .02). Non-statistically significant trends suggested that referred patients had longer hospital length of stay (9.4[7.1] days, compared with 5.4[4.6] days in not referred group), as well as more hospital bed days in the last 3 years (36.4[27.6] days compared with 18.3[23.2] days in not referred group). There was a trend for referred patients to have been seen by the COPD coordinator (100%, compared with 50% in not referred group), and to have previously discussed pulmonary rehabilitation with staff on at least one occasion (100%, compared with 60% in not referred group).

Availability of transport (40% in referred, 50% in not referred), and having been seen by a physiotherapist during admission (80% in both groups), did not differ between referred and not referred patients.

As pulmonary rehabilitation was identified as the least well implemented recommendation, barriers and facilitators in relation to its implementation have been reported.

### Barriers and facilitators to pulmonary rehabilitation referral: patient perspectives

The frequency of barriers and enablers described by the participants with regard to pulmonary rehabilitation is described in Table 3. Because participants’ experience of pulmonary rehabilitation was found to be limited, participants were also asked specifically about their experiences of barriers to and facilitators for exercise (a key component of pulmonary rehabilitation).

**Table 3:** **Frequency of patient**- **reported barriers and enablers to participation in pulmonary rehabilitation and exercise**

**Theme**		**Frequency of reporting(from total participants n** = **15)**	
		<25% (up to 2 participants)	25-49% (3–7 participants)
Pulmonary rehabilitation	Barriers	Too exhausting (2/15)	Lack of awareness (3/15)
		I don’t need it (2/15)	Difficulty with access (5/15) (i.e. nuisance to get there, inflexible timing, transport)
		Managing co-morbidities (2/15)	
		Interruption due to ill health (1/15)	
		Competing responsibilities (1/15)	
	Facilitators	Belief in health consequences (1/15)	Social influence (3/15) (peers, family and health professionals)
		Enjoyment (1/15)	
		Learned new skills (1/15)	
Exercise	Barriers	I don’t need it (1/15)	Managing comorbidities (7/15)
		Access (1/15)	
		Interruption due to ill health (1/15)	
		Social influence (1/15)	
	Facilitators	Belief in own capabilities to exercise (2/15)	Belief in positive health consequences (5/15)
			Social influence (3/15)
		Enjoyment (1/15)	
		Flexible timing (1/15)	
		Already linked to this network (2/15)	Motivated/determined to do it (6/15)
		Has the necessary equipment (1/15)	
		Has transport (1/15)	
		Good weather (2/15)	
		Low cost (1/15)	

***Lack of awareness*** was a significant barrier, as indicated by this response to the investigator’s description of pulmonary rehabilitation:

"Another area that sometimes the doctors talk to people with lung problems about is joining in something called pulmonary rehabilitation, or a group to go and learn to do a bit more, get a bit fitter, or get a bit more exercise, or get on a bit better with things. Has anyone ever talked to you about that sort of thing? (investigator)"

"No, I wouldn’t know what that’s about. (id 3)"

***Hearing about the benefits of pulmonary rehabilitation*** from friends, family or health professionals was the most common facilitator of participation.

"I’ve – well, when I was home I decided that I should be able to do some of these things, you know, and there were people that I knew, had emphysema, that joined…., that did all these exercises from different stages they were with their emphysema, and they would assess you, and see how you got on with it. Well I decided, “Yeah. I could do that.” (id1)"

In contrast to the lack of awareness about pulmonary rehabilitation, ***belief in the health benefits of exercise*** was frequent:

"I’m moving around the house all the time, and I’ve got a garden and I get out there and muck around, and all that stuff. I’m continuously bending over, or moving. So I’m not sitting watching television…because it makes me feel better. Rather than just sitting down all the time. (id7)"

***Coping with co***-***morbidities*** was commonly expressed as a barrier to exercise participation, for example in this woman with co-existent chronic back pain:

"Yes, I've got my walker and I can go a little distance and then I flop over which is - and have a rest and then get going again and I just use my walking stick mostly inside because I've got not very big area to walk."

"And is it your back or your lungs that trouble you most with walking? (investigator)"

"Back. (id 13)"

### Barriers and facilitators to pulmonary rehabilitation referral: doctor perspectives

Medical practitioners who participated in this study were general medicine registrars (n = 5) and interns (n = 4), of whom three had previous experience on respiratory medicine rotations. Medical practitioners’ experience of referring people to pulmonary rehabilitation was generally ***infrequent***, citing they had referred none, one or two COPD patients during a three month rotation in general medicine wards. Patients with ***more severe illness***, already on maximal therapy or frequent attenders to hospital were most likely to be referred.

"Last year when I was doing general medicine we certainly had one patient who was just a frequent flyer and would just keep coming in with acute exacerbation ….and he was one where you know to stop him from returning to hospital all the time that we enrolled him into the program. (registrar id3)"

***Previous experience*** in thoracic outpatient clinics or rehabilitation facilitated doctors in making referrals to pulmonary rehabilitation. Doctors who had successfully referred patients described the importance of explaining to the patients how pulmonary rehabilitation would help them to cope with dyspnoea and avoid future hospitalisations.

"I think from my experience a majority of them, and every one that I’ve asked them to go to rehab have agreed. For example Mr B____, he had rehab prior to he went last time, and he wants to go back again. So he’s been enrolled and he’s going."

"What do you think contributes to your success?(investigator)"

"I think the explanation really, and all the patients don’t want to come back with exacerbation of COPD and they hate the hospital environment. (registrar id2)"

***Low awareness*** of the program limited doctors from making more referrals to pulmonary rehabilitation:

"Are there processes within the hospital that makes it easy to suggest pulmonary rehab? (investigator)"

"I think it is something that is not out there openly that we forget to be able to refer to that so I think if it was more publicised and we had more awareness of these things being around that I think we would probably refer more people. (registrar id3)"

Suggestions offered by doctors to improve implementation included raising awareness of the program, streamlining the referral process, and having physiotherapists more actively identify suitable patients to the medical teams.

## Discussion

This study provides a snapshot of the care of consecutive COPD patients admitted to hospital, indicating good compliance with most high-evidence care recommendations. However, referral to pulmonary rehabilitation appears underutilised in comparison, and on the basis of this pilot research warrants investigation in a larger observational study. Such a study appears highly feasible in this centre, with anticipated recruitment rates in patients and medical practitioners able to be predicted from our data.

An Australian study [15] in COPD hospital inpatients found only four of 45 participant (8.9%) had ever completed a rehabilitation program, and 25 (55.6%) had never commenced one. However, reasons for non-referral were not explored. A strength of our study was the use of multiperspective qualitative interviews [16] to gain an understanding of both patient and doctor views regarding the implementation of pulmonary rehabilitation. These data highlight the barriers and facilitators to service implementation which exist at environmental and organisational levels, as well as clinician and patient knowledge and beliefs.

Patient barriers to participation in pulmonary rehabilitation have previously been identified, but only in people with COPD already referred to, or commenced, a rehabilitation program. Commonly expressed barriers, also described in our data, were difficulties with transport, insufficient perceived health benefit and managing ill-health due to COPD and co-morbidities [17-19]. A positive impact of health professional recommendation on uptake of pulmonary rehabilitation has been identified [20], to which our data adds the influence of peers and family. In contrast, our study highlights the problem of low awareness in the cohort of people with COPD who have never been referred to pulmonary rehabilitation during their disease course. Patients in this study expressed motivation to be active and belief in the health benefits of exercise. However, these attitudes did not translate into their understanding of potential benefits from pulmonary rehabilitation. This highlights the need to ask questions in further qualitative research about barriers and facilitators to participation in “exercise” and “daily activities” as well as “pulmonary rehabilitation” as many people with COPD will never have heard of the latter term. Given the key barriers expressed by patients in the current study, raising awareness of pulmonary rehabilitation in COPD, communicating its potential health benefits, addressing co-morbidities and minimising difficulties with access are all indicated.

A number of doctors in this sample were also unfamiliar with pulmonary rehabilitation and did not often consider it in the usual care of patients with COPD. Doctors were more likely to suggest pulmonary rehabilitation for patients with severe disease and frequent hospital admissions, in agreement with quantitative findings in our sample. Reasons for lack of referral to pulmonary rehabilitation have been examined in general practitioners and practice nurses [21]. Interviews identified lack of available services, lack of time, and a perceived difficult referral process, the latter reason in common with our findings. While a lack of services did not emerge in our data as a reason for non-referral, this may have been overshadowed by a general lack of awareness of pulmonary rehabilitation. Further qualitative research in medical practitioners is indicated in this area, and should include specialist as well as junior staff. Specific interview questions to distinguish between barriers related to capability (e.g. awareness/knowledge), motivation (e.g. beliefs about consequences, role) and opportunity (e.g. structural organisation) will help direct interventions to improve implementation [20].

Data in this study are limited to the perspectives of patients at the end of a hospital admission, and doctors caring for COPD patients in a tertiary hospital. However, as COPD is reportedly under-diagnosed in primary care [22], hospital admissions may represent sentinel events in the disease trajectory which initiate diagnosis and guide decisions about future disease management [23]. Hospital admissions thus present critical opportunities for health professionals to positively influence patient management.

Barriers identified in this preliminary study indicate the relative complexity of behaviour change required to implement pulmonary rehabilitation is greater than many other COPD care recommendations, thus likely to present greater challenges to both patients and practitioners. For example, implementation of influenza vaccination requires adoption of a simple, annual procedure, supported by a widespread public information campaign and decision support for general practitioners. Smoking cessation is a more complex behaviour change, but also has a high degree of community awareness and organisational supports including no-smoking policies. In comparison, pulmonary rehabilitation is unknown in the general community and requires attendance at regular sessions, participation in a home program, and adoption of lifestyle changes.

## Conclusions

This study provides justification for a prospective observational study to determine the incidence of pulmonary rehabilitation referral and attendance in a larger cohort of COPD patients, and indicates low awareness of pulmonary rehabilitation amongst patients and medical practitioners. Further investigation will guide development of targeted strategies at patient, clinician, organisational and community levels to improve implementation of this COPD care recommendation.

## Competing interests

The authors declare that they have no competing interests.

## Authors’ contributions

All authors contributed to design of the study. KJ with the assistance of MY and RA were responsible for data collection. KJ analysed the data. KJ, KG-S and PF contributed to the discussion. All authors read and approved the final manuscript.
